# Sperm and testicular dysfunction during cecal ligation and puncture-induced sepsis in male rats and effects of tannic acid through reducing testicular oxidative stress and inflammation

**DOI:** 10.22038/IJBMS.2021.59375.13183

**Published:** 2021-11

**Authors:** Fatemeh Pourmirzaei, Mina Ranjbaran, Mehri Kadkhodaee, Farzaneh Kianian, Keivan Lorian, Arash Abdi, Mahdi Hajiaghaei, Behjat Seifi

**Affiliations:** 1 Department of Physiology, School of Medicine, Tehran University of Medical Sciences, Tehran, Iran; 2 Department of Reproductive Biology, Shahid Sadoughi University of Medical Sciences, Yazd, Iran

**Keywords:** Inflammation, Oxidative stress, Sepsis, Sperm, Tannic acid, Testicular dysfunction

## Abstract

**Objective(s):**

One of the problems caused by infectious diseases is the decrease in sperm count and motility. Tannic acid is known as an anti-oxidant and anti-inflammatory agent. In this study, Cecal Ligation and Puncture (CLP) sepsis model was induced to investigate the effect of tannic acid on oxidative stress and inflammation in testicular and sperm structure and function.

**Materials and Methods:**

Twenty-four male Wistar rats (250–300 g) were randomly divided into 3 groups of 8: 1) sham, 2) sepsis, and 3) sepsis + tannic acid (20 mg/kg at 6, 12, and 24 hr after sepsis induction). Thirty hours after induction of sepsis, testicular samples were collected to measure SOD activity and MDA, IL-6, and TNF-α levels. Another part of the testis was fixed in 10% formalin for histological examinations.

**Results:**

In the sepsis group, testicular MDA, TNF-α, and IL-6 levels increased and SOD activity decreased compared with the sham group. In addition, the percentage of motile sperm and the survival rate of sperm decreased significantly in the sepsis group. Administration of tannic acid significantly decreased inflammatory markers (TNF-α and IL-6) and MDA levels and increased SOD activity. Furthermore tannic acid significantly improved sperm parameters and increased sperm and animal survival rates.

**Conclusion:**

The results of this study showed that the reproductive system may be strongly affected by the conditions created during sepsis. Tannic acid improved reproductive dysfunction in sepsis by reducing oxidative stress and inflammation.

## Introduction

One of the complications and problems observed in conditions such as sepsis and infectious diseases is a considerable detrimental effect on sperm quality. While there are many causes for male infertility, it is estimated that infectious processes account for about 5% of these cases (1). The association between inflammatory and oxidative processes is one of the pathophysiological mechanisms that occur during sepsis. During sepsis, the body temperature increment can negatively affect epididymal and seminiferous tubules’ function in addition to sperm parameters such as motility, concentration, and morphology. It can be helpful to use a substance that has an anti-inflammatory effect and lowers the body temperature (2). Studies have shown that anti-oxidants protect sperm against reactive oxygen species (ROS) produced by abnormal sperm (3, 4). In this way, they reduce the ROS produced by leukocytes, prevent DNA fragmentation and damage to proteins, improve semen quality and membrane lipid, and increase sperm motility (5, 6). 

In recent years phenolic compounds have found a special place in human health due to their different crucial bioactivities. They are a class of natural anti-oxidants, and tannic acid is one of them. Tannic acid is a plant polyphenolic compound found in tea, coffee, red wine, and unripe fruits. It is soluble in water and has low acidity due to its abundant phenolic functional group (7). Testis with high levels of fatty acid are vulnerable to lipid peroxidation and production of ROS and increased oxidative stress could have a notable effect on testicular dysfunction and sperm parameters (8). Tannins with anti-oxidant and anti-free radical properties are probably useful for animal reproduction (9). Their functions include anti-anti-oxidant activity, modification of lipid peroxidation processes, inhibition of hydrogen peroxide, reduction of iron-oxidizing power with chelating properties, and anti-inflammatory effects (10, 11). Tannins and tannic acid have been shown to affect sperm function by suppressing free radical scavenging properties and improving reproductive organ weight, serum sex hormone, and autophagy in the testis, and they ameliorate sperm parameters and aberration in the chromosome (9). This study aimed to evaluate the protective effect of tannic acid on sperm function, testicular structure, oxidative stress, and inflammation caused by Cecal Ligation and Puncture (CLP)-induced sepsis in male rats. 

## Materials and Mrthods

In this study, twenty-four male Wistar rats weighing 250–300 g were used. Animals were kept in the animal laboratory in a controlled environment, a cycle of 12 hr of darkness-light, and 22 ± 2 °C temperature, with free access to food and water. All experiments were approved by the Ethics Committee of Tehran University of Medical Science. (IR.TUMS.MEDICINE.REC.1398.821). 

Animals were randomly assigned into 3 groups of 8: 1- Sham, 2- Sepsis, and 3- Sepsis + tannic acid. 

Sham group: In this group, animals underwent anesthesia and surgery, but sepsis was not induced and after 30 hr, animals were anesthetized again and sampling was performed.

Sepsis group: In this group, after induction of sepsis by the CLP method, animals were kept for 30 hr, then anesthetized and sampling was performed.

Sepsis + tannic acid group: In this group, animals received intraperitoneal injection of tannic acid at a dose of 20 mg/kg 6, 12, and 24 hr after sepsis induction, and sampling was performed 30 hr after sepsis induction. 

Animals in the sham and sepsis groups received intraperitoneal injection of ethanol as vehicle at 6, 12, and 24 hr after surgery.

***Sepsis induction by Cecal Ligation and Puncture Mo*** **del (CLP)**

The animals were anesthetized with ketamine (70 mg/kg) and xylazine (10 mg/kg) and then underwent intermediate laparotomy. The abdominal viscera were transferred to one side of the body, and the cecum was pushed out of the abdominal cavity. The connective tissue between the cecum and small intestine was removed gently with two moistened ear swabs to prevent damage to the blood vessels. Then 30 to 40% of the end of the cecum was sutured in two layers with 4-0 silk suture. After that, the perforations were made in the cecum area with a 25-gauge needle, and with a little pressure, the small amount of feces were extruded into the peritoneal cavity. The intestine and cecum were carefully returned to their place. The abdominal incision place was sutured in two layers (muscle and skin) with 4-0 silk sutures. After surgery, the suture site was covered with tetracycline antibiotics. For fluid resuscitation, 3 ml of normal saline per 100 g were injected subcutaneously in animals. After 6 hr, ketorolac at a dose of 0.86 mg/kg was injected intramuscularly in all animals. Then, 30 hr after surgery, the animals were anesthetized again and sampling was performed (12).

***Determination of the drug dose***

To determine the appropriate dose and injection time of tannic acid, different doses and times were evaluated and the best one was selected.

Twenty mg of tannic acid powder was added to 1 ml of 10% ethanol and completely dissolved using a shaker. The solution was freshly prepared and stored in a sealed container at room temperature and away from light.

***Assessment of systolic blood pressure***

Systolic blood pressure was measured using a tail-cuff method one hour before sampling. Systolic blood pressure was recorded three successive times. A difference of less than 5 mmHg was considered average pressure (3).

***Assessment of arterial blood oxygen saturation***

One hour before beginning of sampling, the percentage of arterial blood oxygen saturation was assessed by pulse oximetry. 

***Assessment of body temperature***

To measure body temperature, the animals were anesthetized, and a thermometer was placed inside the rat’s anus.

Systolic blood pressure, arterial blood oxygen saturation percentage, and body temperature were measured to ensure induction of the sepsis model.

***Sampling of testicular tissue***

The right testicular tissue was used to measure SOD activity and MDA, TNF-α, and IL-6 levels. The left testicular tissue sample was fixed in 10% formalin for histological examination.

***Assessment of testicular oxidative stress markers (Malondialdehyde (MDA) level and superoxide dismutase (SOD) enzyme activity)***

MDA levels in testicular tissue were measured by Esterbauer and Cheeseman methods and based on their reaction with thiobarbituric acid (TBA). MDA reacts with TBA to produce a pink pigment with maximum absorption of 532 nm (13).

Nasdox ELISA kit was used to measure the activity of the superoxide dismutase enzyme. All ELISA kit contents and samples were placed at room temperature. For 100 mg of testicular tissue, 500 μl of lubricating buffer was added and homogenized. The solution was then centrifuged at 12,000 rpm for 5 min at 4 °C and the supernatant was taken to measure SOD activity. First, 50 μl of samples were added to all microtiter plate wells. Deionized water (50 microliters) was added to the control wells. In the next step, 200 μl of R1 reagent and then 50 μl of R2 reagent were added to all wells. After 5 min of incubation at room temperature and darkness, the light absorption of the samples was read using an ELISA reader at 405 nm. Finally, the level of enzyme activity was calculated using the following formula:

SOD activity (U/ml or mg protein) = OD Test/OD Control × 200

***Assessment of inflammatory markers in testicular tissue by Western blotting (TNF-α and IL-6 levels)***

The level of inflammatory cytokines in testicular tissue was measured by Western blotting (14).

The testicular tissue of rats was homogenized in lysis buffer (50 mM Tris–HCl pH 7.5, 137 mM NaCl, 0.5% Triton X-100, EDTA-free 1× complete protease inhibitor mixture, Roche, Massachusetts, USA) on ice, then sonicated, and incubated on ice for 15 min before centrifugation at maximum speed in an Eppendorf centrifuge at +4 °C for 10 min to isolate lysate. Protein concentrations of cell lysates were determined by Bradford’s method. A standard plot was generated from bovine serum albumin. Then, the total proteins were electrophoresed in 12% SDS-PAGE gels, transferred to polyvinylidene fluoride membranes and probed with Bax and Bcl-2 antibodies, and then probed with secondary antibody. Chemiluminescence was done using an enhanced electrochemiluminescence reagent kit and subsequent autoradiography detection was by immunoreactive polypeptides. The results were performed quantity using densitometry scan of the films. The PVDF membranes were stripped and reused using an anti-actin antibody to normalize protein loading and transfer. Data analysis was performed by Image J, then the integrated density of bands was measured after background subtraction.

***Sperm preparation***

The sperm washing media was pre-warmed to 37 °C in the incubator. The caudal part of the epididymis was minced and incubated in 5 ml of the media for 15 min.

***Evaluation of sperm parameters***

*Sperm motility*

To determine the sperm motility, 10 μl of sperm suspension was placed on prewarmed slides, and motility of 200 sperm was examined in 10 microscopic fields with 400 magnification. The average was recorded as the percentage of sperm motility according to the new WHO classification (15). Progressive motile sperm (PMS): Progressive and active movement regardless of speed, Non-progressive motile sperm (NPMS): All types of movement without progressive movement, Non-motile sperm (NMS): No movement.

*Sperm viability*

Eosin-nigrosin staining was used according to the WHO recommended protocol. 1% eosin and 10% nigrosin were prepared in distilled water, one volume of sperm suspension with two volumes of 1% eosin was mixed, and an equal volume of nigrosin was added to the mixture after 30 sec. Live sperm remained stainless, and dead sperm became pink. The viability of 100 sperm at 100 magnification was assessed by using a light microscope. The percentage of sperm viability was recorded. 

*Histological procedure*

Testicular tissue was fixed in 10% formalin and embedded in paraffin. Tissue sections were prepared with a thickness of 4 μm and stained with hematoxylin-eosin. They were examined by light microscopy. The slides were examined by two independent observers. The changes in seminiferous tubules (tissue rupture, interstitial tissue degeneration, discharge of tubes from germ cell lineage, distance between seminiferous tubules, and discharge of tubes from cells), basement membranes, and the surrounding interstitial tissues were evaluated.

***Statistical analysis***

Data were expressed as mean±SEM. One-way analysis of variance followed by Tukey’s *post hoc* was used to compare different groups. *P*<0.05 was considered statistically significant. 

## Results

***Effect of tannic acid on systolic blood pressure in CLP-induced sepsis***

**Figure 1:**
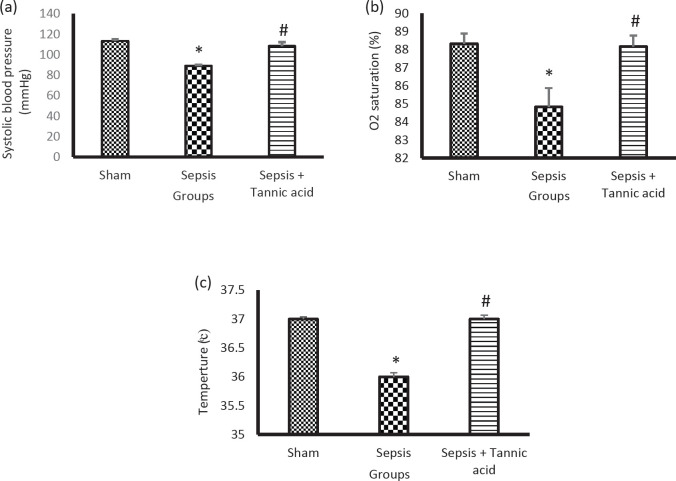
Indices of sepsis induction. (a) Changes in systolic blood pressure in different groups. (b) Changes in arterial blood oxygen saturation percentage in different groups. (c) Changes in body temperature in different groups. * *P*<0.05 significant differences to the sham group. # *P*<0.05 significant differences to sepsis group

Systolic blood pressure was significantly declined to lower levels in the sepsis group compared with the sham group (89 ± 0.96 vs 113.33 ± 1.6 mmHg, *P*<0.05). Administration of tannic acid significantly prevented hypotension compared with the sepsis group (108.3 ± 3.85 vs 89 ± 0.96 mmHg, *P*<0.05, Figure 1a).

***Effect of tannic acid on arterial blood oxygen saturation in CLP-induced sepsis***

Sepsis caused a significant decrease in the percentage of arterial blood oxygen saturation in the sepsis group compared with the sham group (84.83 ± 1.04 vs 88.33 ± 0.55 *P*<0.05). Administration of tannic acid caused a significant increase in arterial blood oxygen saturation compared with the sepsis group (88.17 ± 0.67 vs 84.83 ± 1.04, *P*<0.05, Figure 1b).

***Effect of tannic acid on the body temperature in CLP-induced sepsis***

Sepsis caused a significant decrease in body temperature in the sepsis group compared with the sham group (36 ± 0.072 vs 37 ± 0.04 °C, *P*<0.05). Administration of tannic acid caused a significant increase in body temperature compared with the sepsis group (37 ± 0.06 vs 36 ± 0.07 °C, *P*<0.05, Figure 1c).

***Effect of tannic acid on oxidative stress indices in CLP-induced sepsis***

**Figure 2:**
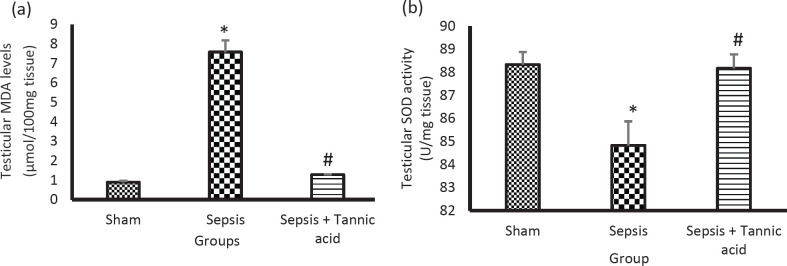
Effect of tannic acid on oxidative stress indices in CLP-induced sepsis. (a) Changes in MDA levels in different groups. (b) Changes in SOD enzyme activity in different groups. * *P*<0.05 significant differences to the sham group. # *P*<0.05 significant differences to sepsis group

Sepsis caused a significant increase in the testicular MDA levels in the sepsis group compared with the sham group (7.58 ± 0.59 vs 0.89 ± 0.07 µmol/100 mg tissue, *P*<0.05). Administration of tannic acid reduced MDA levels compared with the sepsis group (1.28 ± 0.01 vs 7.58 ± 0.59 µmol/100 mg tissue, *P*<0.05, Figure 2a).

Sepsis caused a significant decrease in the activity of the SOD enzyme in testicular tissue in the sepsis group compared with the sham group (24.21 ± 1.95 vs 69.5 ± 1.79 U/mg tissue, *P*<0.05). Administration of tannic acid increased the activity of the SOD enzyme compared with the sepsis group (68.25 ± 1.99 vs 24.21 ± 1.95 U/mg tissue, *P*<0.05, Figure 2b).

***Effect of tannic acid on inflammatory markers of testicular tissue in CLP-induced sepsis***

**Figure 3:**
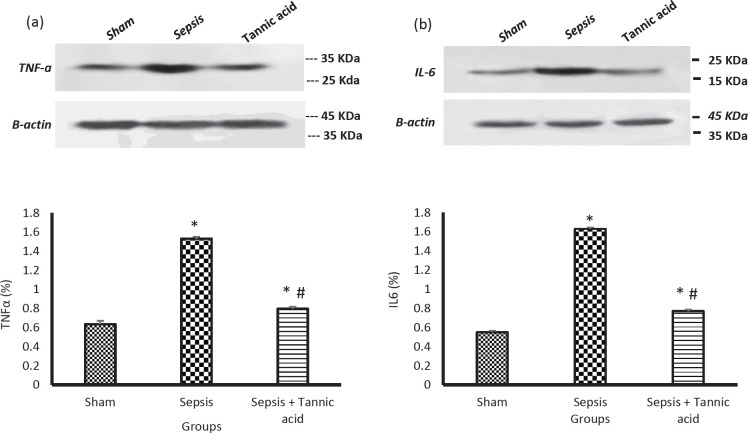
Effect of tannic acid on inflammatory markers of testicular tissue in CLP-induced sepsis. (a) Changes in TNF-α protein expression in testicular tissue in different groups. (b) Changes in IL-6 protein expression in testicular tissue in different groups. * *P*<0.05 significant differences to the sham group. # * P*<0.05 significant differences to sepsis group

Sepsis caused a significant increase in the expression of TNF-α level in testicular tissue in the sepsis group compared with the sham group (1.53 ± 0.02 vs 0.63 ± 0.03 %, *P*<0.05). Administration of tannic acid reduced the level of TNF-α in testicular tissue compared with the sepsis group (0.79 ± 0.02 vs 1.53 ± 0.02 %, *P*<0.05, Figure 3a).

Sepsis caused a significant increase in the expression of IL-6 level in testicular tissue in the sepsis group compared with the sham group (1.65 ± 0.01 vs 0.55 ± 0.01 %, *P*<0.05). Administration of tannic acid decreased the level of IL-6 in testicular tissue compared with the sepsis group (0.77 ± 0.01 vs 1.65 ± 0.01 %, *P*<0.05, Figure 3b). 

***Effect of tannic acid on sperm parameters in CLP-induced sepsis***

*Effect of tannic acid on progressive motile sperm (PMS)*

**Figure 4:**
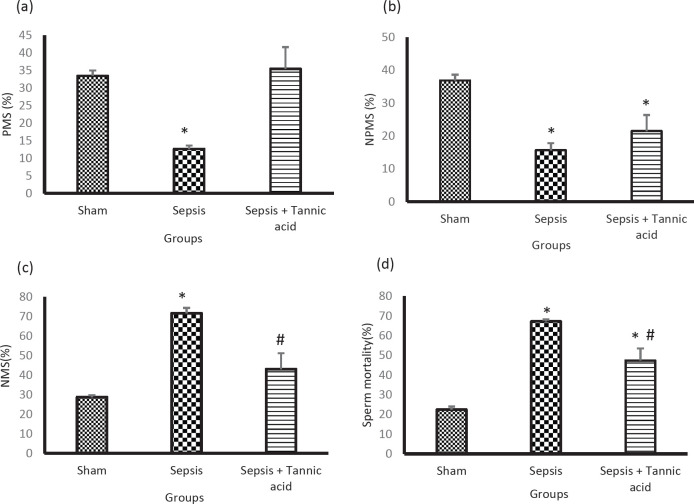
Effect of tannic acid on sperm parameters and sperm viability in CLP-induced sepsis. (a) Percentage of sperm with progressive movement in different groups. (b) Percentage of non-progressive motile sperm in different groups. (c) Percentage of immobile sperm in different groups. (d) Percentage of sperm mortality in different groups. * *P*<0.05 significant differences to the sham group. # *P*<0.05 significant differences to sepsis group

Sepsis caused a decrease in the number of sperm with progressive movement in the sepsis group compared with the sham group (12.66 ± 0.95 vs 33.5 ± 1.52 %, *P*<0.05). Administration of tannic acid increased the number of sperm with progressive movement compared with the sepsis group (35.5 ± 6.2 vs 12.66 ± 0.95 %, *P*<0.05, Figure 4a).

*Tannic acid did not cause a significant change in non-progressive motile sperm (NPMS)*

Sepsis reduced the number of NPMS in the sepsis group compared with the sham group (15.66 ± 2.17 vs 36.91 ± 1.79 %, *P*<0.05). Administration of tannic acid did not change the number of NPMS compared with the sepsis group (21.5 ± 4.86 vs 15.66 ± 2.17 %, *P*<0.05, Figure 4b)***.***

*Tannic acid decreased non-motile sperm (NMS)*

Sepsis increased the number of immobile sperm in the sepsis group compared with the sham group (71.66 ± 2.7 vs 28.75 ± 0.98 %, *P*<0.05). Administration of tannic acid reduced immobilized sperm compared with the sepsis group (43 ± 8.15 vs 71.66 ± 2.7 %, *P*<0.05, Figure 4c)***.***

*Effect of tannic acid on sperm viability*

Sepsis increased the number of dead sperm in the sepsis group compared with the sham group (67.24 ± 0.95 vs 22.5 ± 1.52 %, *P*<0.05). Administration of tannic acid reduced the number of dead sperm compared with the sepsis group (47.33 ± 6.25 vs 67.24 ± 0.95 %, *P*<0.05, Figure 4d)***.***

*Effect of tannic acid on testicular histology in CLP-induced sepsis*

**Figure 5:**
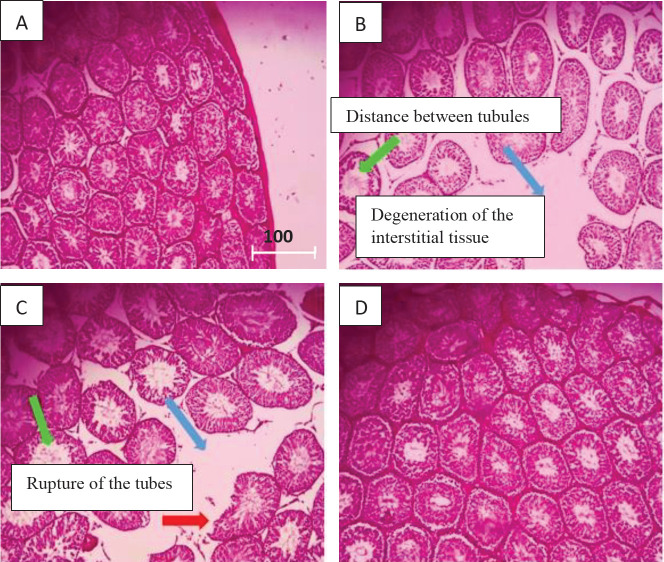
Testicular histology in different groups. A) Sham, B) Sepsis, C) Sepsis, and D) Sepsis+tannic acid. In the sham group (A) the testis tissue was intact. In the sepsis group (B and C) tissue rupture, interstitial tissue degeneration, discharge of tubes from germ cell lineage, distance between seminiferous tubules, and discharge of tubes from cells are observed. Administration of tannic acid (D) improved tissue damage. Red arrow, blue arrow: degeneration of the interstitial tissue and the distance between the seminiferous tubules. Green Arrow: empty tubes from the cells. Magnification: 200%. Resolution 600 dpi

The tissue sections of the sham groups (A) were almost normal. The tubules were arranged regularly. Sepsis caused changes in the testicular tissue of the sepsis group (B and C) compared with the sham group. There was rupture of the tubules and wrinkles around the testicular tissue and drainage of the tubules from Sertoli cells and spermatogonia in the sepsis group. Administration of tannic acid improved the structural changes of testicular tissue compared with the sepsis group (Figure 5).

## Discussion

The main focus of the present study was to elucidate the effects of natural polyphenol tannic acid on sperm and testicular dysfunction during CLP-induced sepsis. The present study showed that sepsis as a causative agent of infection leads to an imbalance between anti-oxidants and production of ROS and inflammatory conditions in testicular tissue. Administration of tannic acid increased the survival rate of animals in the treatment group (87.5%) compared with the sepsis group (38.09%) which indicates the positive effect of tannic acid on improving infectious conditions (data not shown). Studies showed pretreatment with tannic acid in infectious diseases enhances the epididymal function by increasing sperm count, motility and decreasing sperm morphology aberration (16). Moreover, the tannic acid was administrated to animals alone and results were similar to the sham group. None of the parameters especially sperm parameters did alter compared with the sham group (data not shown). The present study finding is similar to mentioned study and demonstrates that plant polyphenol is able to improve sperm parameters and morphology.

The effects of acute infection on male health and fertility should be taken into consideration, specifically stress and infection that lead to an increase in corticosteroid and cytokine levels which contribute to the inflammatory process. High secretion of cortisol may reduce testosterone. Testosterone is an essential hormone for spermatogenesis, thus this reduction could theoretically restrict the development and maturation of spermatogonia (17). 

In addition, it was observed that administration of tannic acid as an anti-inflammatory agent reduced the levels of inflammatory cytokines, TNF-α and IL-6, in sepsis as it was observed in bone marrow-derived macrophages cells (11). Therefore by reduction in the inflammatory and oxidative stress created during sepsis, the negative effects of these factors were neutralized on sperm motility and survival to a large extent which ultimately leads to better conditions. 

Karuppagounder *et al*. reported tannic acid reduced inflammatory mediators, cyclooxygenase (COX)2, and inducible nitric oxide synthase (iNOS). Inhibition of inflammatory factors by tannic acid might be mediated through NFκB signaling pathway (18). 

During sepsis, some disturbances may happen in the cardiovascular system. Tannic acid may cause a positive effect on the arterial blood oxygen saturation, reducing hemodynamic abnormalities in the circulatory system and pulmonary function through lowering the inflammatory cytokines (19, 20). The hypoxia observed during sepsis may lead to spermatogenesis disturbance and deleterious effects on Leydig and Sertoli cells. In the present study tannic acid administration had positive effects on testicular tissue by increasing the arterial blood oxygen saturation.

In the present study, the body temperature had a significant decrease compared with the sham group 28 to 29 hr after induction of sepsis. To justify this, it should be noted that a short time after the onset of sepsis, the animals enter a severe septic phase, if the treatment is not successful. The initial proinflammatory state of sepsis is often suppressed by a prolonged state in which the immune system is restored and then reducing the immune system’s response to inflammatory cytokines (21, 22). It seems that the administration of tannic acid in septic animals showed a more balanced effect on body temperature by reducing the levels of inflammatory cytokines.

In the present study, the protective effect of tannic acid was observed in improving the oxidative stress status of testicular tissue, including increasing SOD enzyme activity and decreasing MDA levels as it has been reported in the pulmonary cells (10). Studies have shown that physiological levels of ROS in the male reproductive tract are essential for proper sperm function, capacity regulation, and spermatozoon response to oocytes. In addition, it was reported a pathological increment of ROS has been associated with male infertility (23). In this study, it was observed that tannic acid improved stress status (MDA level and SOD activity) and decreased tissue oxidative stress damages via anti-oxidative effects. The increased levels of MDA may affect sperm fertility and motility (24). It was reported, spermatozoa may affect mainly in three ways if the oxidants outnumber anti-oxidants: lipid peroxidation, DNA damage, and induction of apoptosis (25). It was shown that tannic acid has actions such as an anti-oxidant effect, improving DNA damage, and inhibiting hydroxyl radical formation (26). Skena *et al*. showed that tannic acid potentially decreased environmental toxicity and improved reproductive health via amelioration of the nucleic acid levels, up-regulating the expression of anti-oxidant Mrna, and alleviating apoptosis by modulating Bax and Bcl2 expression in testicular cells (16). Researchers in 2021 showed that tannic acid suppresses reactive oxygen species-mediated endoplasmic reticulum stress in H9C2 cell apoptosis (27).

By increasing the oxidant levels, cell membranes are more vulnerable to lipid peroxidation. Thus testicular injuries and an increase in the weight of testis may be observed. Mašek *et al*. showed that tannic acid administration decreased lipid peroxidation, increased monounsaturated fatty acids (MUFAs), and decreased testis weight in rats (28). However, increased levels of MUFA can reduce oxidative stress conditions, testis with high levels of polyunsaturated fatty acids (PUFAs) exhibits more sensitivity to oxidative damage (29).

PMS: progressive motile sperm, NPMS: non-progressive motile sperm, NMS: non-motile sperm

## Conclusion

Taken together, the present study suggests CLP induced-sepsis may cause sperm and testicular dysfunction, and presented data show that treatment with tannic acid ameliorates oxidative and inflammatory conditions in testicular tissue. This study suggests that tannic acid administration may be a hopeful therapeutics strategy for sperm and testicular dysfunction in sepsis.

## Authors’ Contributions

BS and MR conceived and designed the research. FP Conducted the experiments. FK, AA, KL, and MH contributed to indices assessments. BS analyzed data. FP and BS wrote the manuscript. MK edited the manuscript.

## Funding

This research was supported by a grant from Tehran University of Medical Sciences (no#98-3-101-45643).

## Conflicts of Interest

There are no conflicts of interest in this study.
